# The function of p97/valosin-containing protein (VCP) and small VCP-interacting protein (SVIP) in invasion and migration of pancreatic cancer cells

**DOI:** 10.55730/1300-0144.5894

**Published:** 2024-06-04

**Authors:** Ebru ALİMOĞULLARI, Sevil ÇAYLI

**Affiliations:** Department of Histology and Embryology, Faculty of Medicine, Ankara Yıldırım Beyazıt University, Ankara, Turkiye

**Keywords:** Endoplasmic reticulum-associated protein degradation (ERAD), invasion, migration, MIA PaCa-2, PANC-1, p97/valosin-containing protein (VCP), small VCP-interacting protein (SVIP)

## Abstract

**Background/aim:**

Misfolded proteins are eliminated by a process known as endoplasmic reticulum-associated protein degradation (ERAD). ERAD has an impact on a variety of illnesses, such as diabetes, cystic fibrosis, cancer, and neurological conditions. As one of the many proteins involved in ERAD, this study is focused on p97/valosin-containing protein (VCP) and small VCP-interacting protein (SVIP). The existence and function of SVIP and p97/VCP in various types of pancreatic cancer have not yet been investigated. The study’s objectives are to examine the expressions of SVIP and p97/VCP in two pancreatic cancer types and to show whether these proteins aid in the invasion and migration of pancreatic cancer cells.

**Materials and methods:**

In this work, MIA PaCa-2 and PANC-1 human cell lines were examined. Immunocytochemistry and immunofluorescence were performed to detect the cellular localization and presence of p97/VCP and SVIP in pancreatic cancer cells. Following p97/VCP siRNA and SVIP siRNA transfection of the cells, protein expressions were assessed using Western blot analysis. The effects of this suppression on cell invasion and migration were determined using the xCELLigence real-time analysis system (RTAC).

**Results:**

In the nucleus and cytoplasm of MIA PaCa-2 and PANC-1 cells, p97/VCP and SVIP immunoexpressions were seen. The decrease in protein expressions of p97/VCPsi and SVIPsi was significant in pancreatic cells compared to the controlsi. The p97/VCP siRNA transfection reduced the invasion and migration of MIA PaCa-2 and PANC-1 cells. In addition, the SVIP siRNA suppression resulted in increasing the invasion and migration ability of both cells. This study also demonstrated, for the first time, SVIP expression in MIA PaCa-2 and PANC-1 cells.

**Conclusion:**

Overall, the findings show the differential expression and function of p97/VCP and SVIP in pancreas ductal adenocarcinoma cells. The potential of the pancreatic cancer cells to migrate and invade altered when the two cell lines were transfected with p97/VCPsi and SVIPsi.

## 1. Introduction

Pancreatic ductal adenocarcinoma (PDAC), a highly malignant disease, makes up 90% of all pancreatic cancers, has a five-year survival rate of 8% over all stages, and is the fourth greatest cause of cancer-related deaths worldwide [1,2]. The primary reasons for mortality from cancer are high invasion and metastasis. Most patients have already missed their chance for surgical resection and efficient therapeutic treatment at the time of diagnosis. To enhance the survival of patients and the quality of life, it is necessary to comprehend the processes of pancreatic cancer that underlie its high propensity for metastasis and to create effective treatments that target metastasis [3–6].

Pancreatic cancer originates from either exocrine or endocrine cells, and exocrine and endocrine tumors are different histologically. Endocrine tumors are generally less common and account for 5% of all pancreatic cancers [7]. Pancreatic cancers consisting of exocrine cells are divided into two main histological subgroups. The pancreatic ductal adenocarcinoma (PDAC) subgroup constitutes the vast majority of exocrine tumors. PDAC originates from epithelial cells such as pancreatic ducts and glands [8]. These cells are usually capable of metastasis to the lymph nodes and liver [9]. It has been indicated that invasive PDAC develops from noninvasive precursor lesions [10].

Misfolded proteins in the endoplasmic reticulum (ER) are degraded by a process called endoplasmic reticulum-associated protein degradation (ERAD), which starts with the recognition of misfolded proteins and ends with proteasome degradation [11]. ERAD has a function in the pathogenesis of numerous illnesses such as diabetes, neurodegenerative disease, cancer, and cystic fibrosis [12].

The 97-kDa valosin-containing protein (p97/VCP) has important functions in ubiquitin-proteasome system (UPS)-dependent proteolysis. VCP acts as a chaperone protein in UPS. It is known that more than 1% of the total cellular protein is VCP [13,14]. p97/VCP is included in many cellular events, involving mitophagy [15], degradation of polyubiquitinated proteins in proteasome [16,17], ubiquitination of misfolded proteins from the ER [18–20], cell cycle regulation [21], and activation of transcription factors [22,23]. In addition, significant changes in p97/VCP expression have been observed in many cancer types, and the use of proteasome inhibitors, such as bortezomib, or p97/VCP inhibitors, such as DBeQ, has been suggested in the treatment of cancer or cancerous tissue. In addition, recent studies have demonstrated that Hrd1 and p97/VCP belonging to the ERAD family are responsible for the destruction of insulin in pancreatic islets [24,25].

Consisting of 76 amino acids, SVIP (Small VCP-interacting protein) is a 9-Kda adaptor endoplasmic reticulum protein that could bind directly to p97/VCP. SVIP is located on the ER membrane’s cytosolic surface. It inhibits the ERAD pathway, which is known independently of ubiquitin, by interacting with p97/VCP, which has a central role in this pathway. Especially for the ERAD pathway, the SVIP protein has been identified as a negative regulator [26]. SVIP was identified as a novel adapter protein for p97/VCP by observing its overexpression in cells, which causes extensive vacuolation and deformation of the ER and microtubules [27].

Endoplasmic reticulum (ER) stress is a significant cellular event that develops as a result of different stimuli. There are different types of responses to ER stress. One of these is the evolutionarily protected unfolded protein response (UPR). The cell may either survive or die as a result of ER stress. [28]. In many studies, ER stress is related with several types of diseases; for example, Alzheimer’s disease, diabetes, prion disease, cancer, and others are shown to be linked to ER stress [29]. Studies have indicated that pancreatic cancer is related to ER stress. Pancreatic cancer has a low curable potential due to its late diagnosis. The secretion of hormones and enzymes is highly functional for pancreatic epithelial cells, so the ER is highly developed. Investigation into the relationship between pancreatic cancer and ER stress is being considered as a new treatment approach. However, although there have been studies on p97/VCP [30], the function, existence, and importance of SVIP in the subtypes of pancreatic cancer have not been examined. In the treatment of pancreatic cancer, combined radiotherapy and chemotherapy treatments are applied to maximally prevent tumor spread. A large number of cytotoxic drugs have been put into practice with different radiation techniques. Recent research has shown that some drugs and substances regulate the spread of tumor tissue through a combination of cell cycling, stimulation of cell death, and suppression of DNA repair. In addition to the prognostic and therapeutic molecular studies, it is important to determine the presence and function of it has been proven to exist in two different tumors (glioma and prostate cancer) SVIP and its interacting protein p97/VCP in pancreatic cancer types. For this reason, we aimed to examine the expression of SVIP and p97/VCP on two ERAD pathway proteins and their efficacy on pancreatic cancer migration and invasion.

## 2. Materials and methods

### 2.1. Gene expression analysis

SVIP and p97/VCP gene expression levels were found in the TNMplot database (http://www.tnmplot.com, accessed 19 February 2024) [31]. TNMplot data was used to analyze the SVIP and p97/VCP gene expressions in pancreatic cancer tumors and in normal tissues.

### 2.2. Cell culture and transfections

The PANC-1 (CRL-1469) and MIA-Paca-2 (CRL-1420) human pancreatic adenocarcinoma cell lines were provided by the ATCC. Pancreatic cells were grown in Dulbecco’s modified Eagle’s medium (Lonza) supplemented with 1% antibiotics (penicillin–streptomycin, Capricorn Scientific) and 10% fetal bovine serum (FBS; Capricorn Scientific). The cells were incubated at 37 °C with 5% CO_2_. Transfections were conducted in 6-well and 12-well plates using Lipofectamine 3000 (Invitrogen, USA) as previously defined [32]. The negative control siRNA (1027281, Qiagen), SVIP siRNA (GS258010, Qiagen), and p97/VCP siRNA (GS7415, Qiagen) were purchased from Qiagen. After 48 h of transfection, the cell lines were fixed in 4% formaldehyde for immunocytochemistry and immunofluorescence. The cells were also lysed for Western blotting. At least three duplicates of each transfection were performed.

### 2.3. Immunocytochemistry

The pancreatic cell lines were seeded in 12-well plates on glass coverslips for immunocytochemical studies. Phosphate-buffered saline (PBS) was used to wash the cells. The cells were fixed in 4% formaldehyde for 20 min. After three washes in PBS, the cells were blocked with a blocking solution (Protein Block, Abcam, ab93697) for 1 h. Then, the primary antibodies p97/VCP (1:500 dilution, ab11433, Abcam) and SVIP (1:50 dilution, HPA039807; Sigma) were applied and left overnight at 4 °C. Next, biotinylated goat antipolyvalent antibody (Abcam, ab93697) was applied for 30 min and washed with PBS. Finally, streptavidin peroxidase (Abcam, ab93697) was added to the cells for 30 min. After three washes, for the final color reveal, the cells were treated with DAB chromogen (Abcam, ab64238), counterstained for 30 s with Mayer’s hematoxylin (Merck, 109249), and then mounted with entellan. The slides were photographed under a light microscope (Olympus BX43).

### 2.4. Immunofluorescence

The cells were seeded in 12-well plates with glass coverslips and fixed in 4 % formaldehyde for 20 min. Coverslips were incubated in a blocking serum following three PBS washes. Then, the primary antibodies p97/VCP (1:500, ab11433, Abcam) and SVIP (1:50, HPA039807, Sigma) were added to the coverslips and left overnight at 4 °C. After incubation, the antibodies Texas red conjugated antimouse secondary antibody (1:600, ab6787; Abcam) and FITC-conjugated antirabbit secondary antibody (1:600, ab6717, Abcam) were applied to the cells and incubated for 1 h. The samples were mounted using a DAPI-containing mounting medium (Abcam). The intracellular localization of the SVIP and p97/VCP proteins was assessed using an Olympus BX53 fluorescent microscope.

### 2.5. Immunoblotting

The PANC-1 and MIA PaCa-2 cell lines were prepared for Western blotting. Cell pellets were resuspended in protease inhibitor (Abcam) with a RIPA buffer (Sigma-Aldrich, USA). A standard Bradford assay was applied to determine protein concentration. Following boiling in the sample buffer, a 40-μg sample of total cellular protein was loaded onto a 4%–12 % Bis-Tris gel (Invitrogen). The semidry transfer was applied onto nitrocellulose membrane. 3% nonfat dry milk in PBS was applied. Determination of SVIP and p97/VCP was performed using the primary antibodies p97/VCP (1:2000, ab11433, Abcam, UK), SVIP (1:700, HPA039807, Sigma), and β-actin (1:3000, ab6276, Abcam) and left overnight. PBS-Tween was used to rinse the membrane three times. Then, the secondary antibodies were treated. Immunodetection was determined using a chemiluminescence substrate (ab133406, Abcam, UK) approach following the manufacturer’s instructions. With the use of a gel documentation system (UVP), the Western blot bands were visualized.

### 2.6. Kinetics of cell migration/invasion with xCELLigence real-time cell analyzer system (RTCA)

Cell migration and invasion were continually monitored using the xCELLigence (RTCA) DP instrument (ACEA Biosciences). With the exception that the two chambers are combined prior to the experiment, the setup is analogous to traditional Transwell plates, with microelectrodes connected to lower part of membrane for impedance-based detection of migrating cells. PANC-1 and MIA PaCa-2 cells transfected with SVIP siRNA, p97/VCP siRNA, and the negative control siRNA were subjected to an RTCA DP assay. The instrument, equipped with a CIM-plate 16 (ACEA Biosciences), contains 16 wells. The top of the membrane was precoated with Matrigel (Corning Matrigel Matrix, 356234), which was used for the invasion experiments. After 4 h at 37 °C, the matrigel was allowed to polymerize. The cells were placed in a FBS-free media. 160 μL of 10% FBS medium was added to fill the lower chamber for the invasion and migration assay. Following the manufacturer’s guidelines, the CIM plate 16 was put in the xCELLigence DP instrument at 37 °C and 5% CO_2_ for 1 h to equilibrate the medium. Following the incubation time, a measuring step was applied as a background signal generated by the cell-free media. After 30 min, cells were plated into the upper compartments. To start the experiment, the two chambers were connected, and the upper compartment was filled with the cells (40,000 per well), which had been preincubated with inhibitors (negative control siRNA, p97/VCP siRNA, and SVIP siRNA) in 100 μL of the serum-free medium. After adding the cells, the CIM plate 16 was kept in the laminar flow hood for 30 min. The migration RTCA assay setup was identical to the invasion setup as described except that the top of the membranes was not coated with a layer of Matrigel. Each condition was run in quadruplicate with a programmed signal detection every 15 m for 30 h in the invasion and migration tests. RTCA software (version 1.2, Roche Diagnostics) was used to perform data acquisition and analysis.

### 2.7. Statistical analysis

Significant differences in p97/VCP and SVIP expression levels in the various cancer tissues were evaluated using the Mann–Whitney U test in the TNMplot database (*p < 0.01). The Western blot and band density specification studies were evaluated using IMAGEJ Version 1.46, with the expression degrees presented as mean ± standard error. Statistical analyses were performed and migration/invasion values were analyzed using the GraphPad Prism 8.4.2 software. Data between groups were checked for significance using a two-way analysis of variance test, with values of p < 0.05 considered significant.

## 3. Results

### 3.1. The mRNA expressions of p97/VCP and SVIP in various cancer types

**Figure 1:**
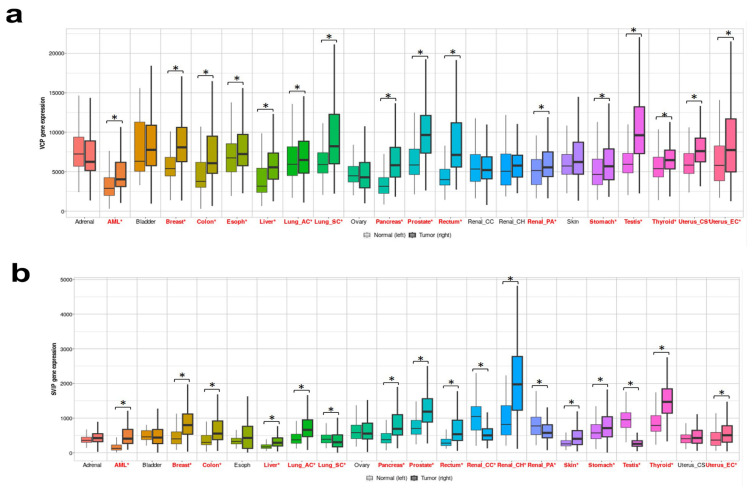
p97/VCP and SVIP expression levels in various human tumors. Decreased or increased expressions of p97/VCP (a) and SVIP (b) in various tumors are shown compared to normal tissues using the TNMplot database. Significant differences according to the Mann–Whitney U-test are highlighted in red (*p < 0.01).

To research the possible differential expressions of p97/VCP and SVIP in normal and tumor tissues, the gene expressions of p97/VCP and SVIP in normal and tumor tissues were compared using the TNMplot database. The expressions of p97/VCP and SVIP mRNA in tumor tissue were clearly higher than in normal tissue for most cancers, including prostate, pancreatic, acute myeloid leukemia, colon, rectal, and breast cancers (Figures 1a and 1b).

### 3.2. Expressions of p97/VCP and SVIP in MIA PaCa-2 and PANC-1 cells

**Figure 2:**
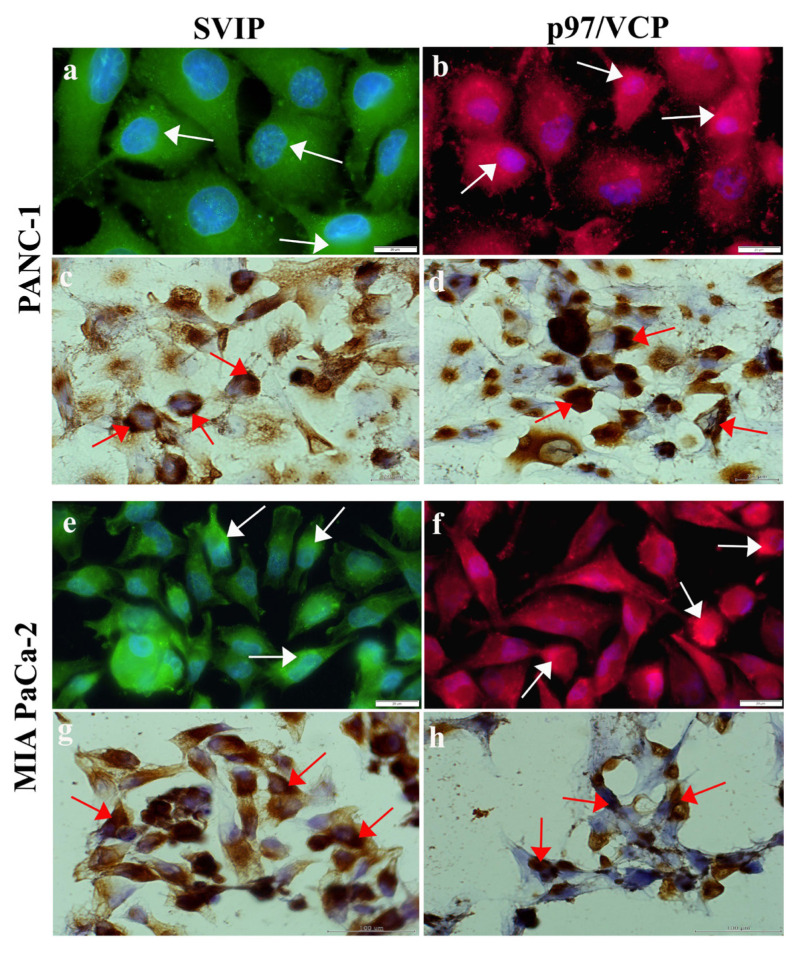
Immunofluorescence (a,b,e,f) and immunocytochemistry (c,d,g,h) staining of p97/VCP and SVIP. Localization of the p97/VCP (arrow) and SVIP (arrow) was determined in the nucleus and cytoplasm of MIA PaCa-2 and PANC-1 cells. Scale bars: 20 μm.

The probable location of p97/VCP and SVIP in pancreatic cancer cell lines is currently unknown. Thus, we identified the cellular localization and immunoexpression of p97/VCP and SVIP in pancreas ductal adenocarcinoma cells. p97/VCP was identified in the nucleus and cytoplasm of MIA PaCa-2 and PANC-1 pancreatic cancer cells (Figures 2b, 2d, 2f, and 2h). The MIA PaCa-2 cells had intense p97/VCP staining in the cytoplasm compared to the PANC-1 cells. In addition, SVIP was identified in the nucleus and cytoplasm of MIA PaCa-2 and PANC-1 pancreas ductal adenocarcinoma cells (Figures 2a, 2c, 2e, and 2g). The MIA PaCa-2 cells had intense SVIP staining in the cytoplasm compared to the PANC-1 cells.

### 3.3. p97/VCP and SVIP expressions in p97/VCP-depleted and SVIP-depleted human pancreatic cells

**Figure 3:**
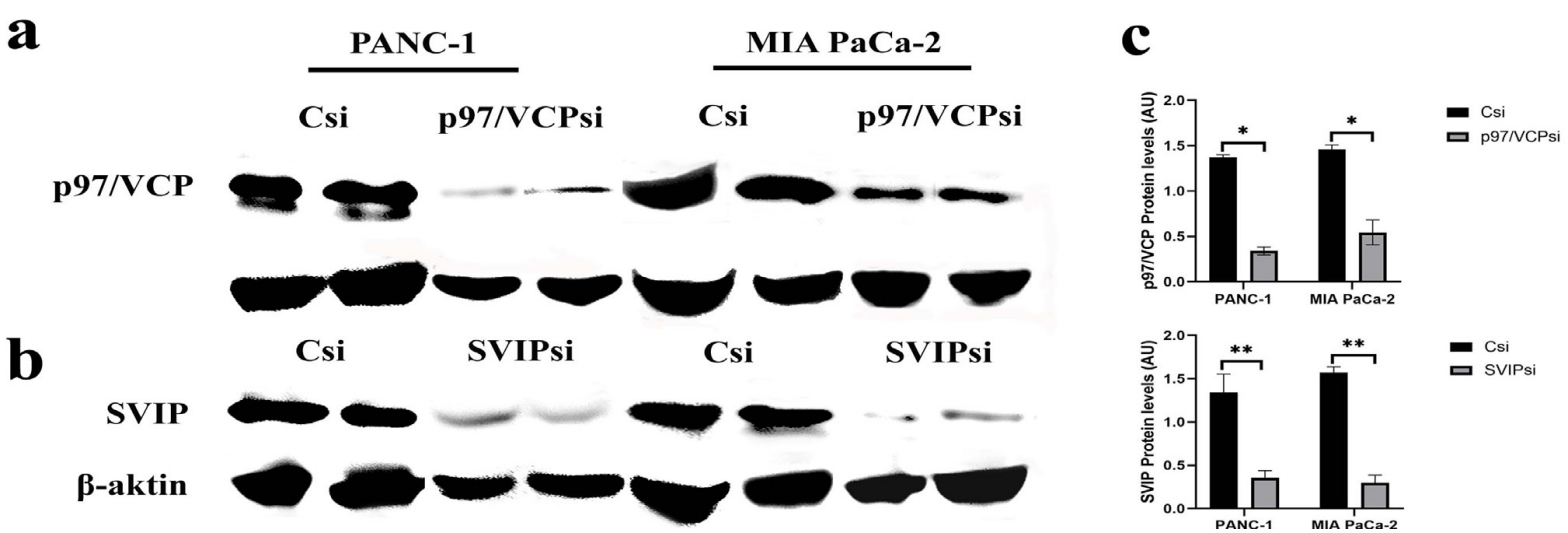
Western blot analysis of the p97/VCP and SVIP proteins following silencing of the expression of p97/VCP and SVIP. The expression of p97/VCP (a) and SVIP (b) proteins, using β-actin as loading control, was evaluated in two pancreatic cells (MIA PaCa-2 and PANC-1). The control siRNA (Csi), VCP-targeted siRNA (p97/VCPsi), and SVIP-targeted siRNA (SVIPsi) were used and treated for 48 h. (c) The silencing of p97/VCP and SVIP expressions significantly decreased p97/VCP and SVIP protein levels in the cells. The densities of the bands were assessed using Image J software. Each bar in the graphs represents mean ± standard error of three independent experiments. *p < 0.05, **p < 0.01.

To further confirm the presence of p97/VCP and SVIP in pancreatic cells, Western blotting was performed in addition to immunocytochemistry and immunofluorescence. The protein expressions of SVIP and p97/VCP were identified in the MIA PaCa-2 and PANC-1 cells based on the Western blot method (Figures 3a and 3b). Pancreatic cancer cells were transiently transfected with SVIP siRNA (SVIPsi), p97/VCP siRNA (p97/VCPsi), and the control siRNA (Csi) to detect whether the protein expressions of SVIP and p97/VCP were influenced by these suppressions. After 48 h of transfection, the p97/VCP protein levels had decreased compared to the control siRNA. Also, the protein expression of SVIP was lower in the SVIP siRNA transfected cells. (Figures 3a and 3b). Furthermore, the decrease in protein expressions of p97/VCPsi and SVIPsi was significant in pancreatic cells compared to the control si (Figure 3c).

### 3.4. The effect of p97/VCP and SVIP on the migration and invasion of pancreatic cancer cells

In view of the potential function of the p97/VCP and SVIP genes in pancreatic cancer progression, the next step was to perform functional analyses. PANC-1 and MIA PaCa-2 cells had their migration and invasion characteristics examined using an xCELLigence real-time cell analyzer system. The experiments were applied in CIM 16-well plates. Migration experiments used wells without Matrigel, but the invasion experiments were performed with matrigel. The cell indices are presented in graphs. Pancreatic cell lines transfected with the control siRNA, p97/VCP siRNA, and SVIP siRNA were then subjected to an RTCA DP assay.

**Figure 4:**
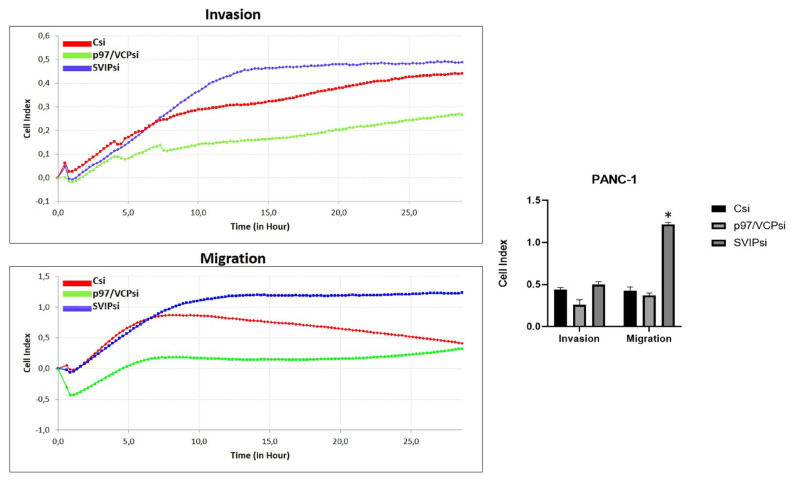
The effects of p97/VCPsi and SVIPsi transfections on cell migration and invasion capacity of PANC-1 cells as measured with xCELLigence RTCA. (a) For 30 h, changes in electrical impedance caused by the cells migrating to the bottoms of the wells were recorded every 15 min. The red curve is the control siRNA transfected cells, the green curve is p97/VCP siRNA transfected cells, and the blue curve is SVIP siRNA transfected cells. (b) Out of minimum three total experiments, each had very similar results, demonstrated by the mean values ± standard deviation of quadruplicate wells. *p < 0.05.

**Figure 5:**
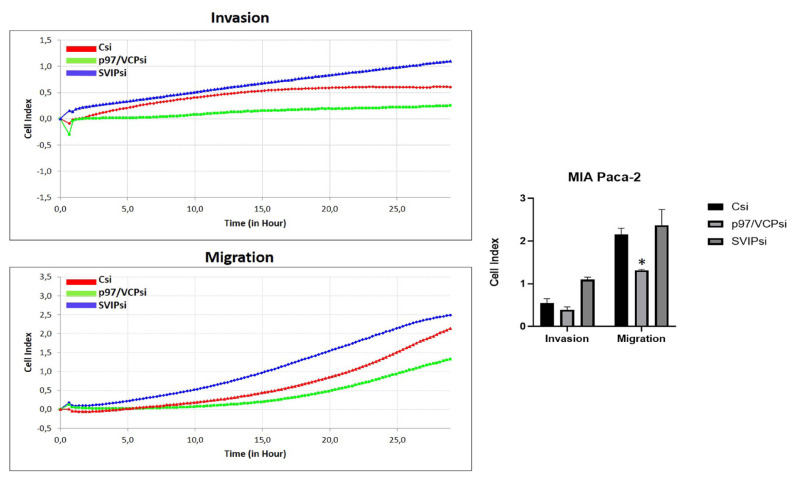
The effects of p97/VCPsi and SVIPsi transfections on cell migration and invasion capacity of MIA PaCa-2 cells as measured with xCELLigence RTCA. (a) For 30 h, changes in electrical impedance caused by cells migrating to the bottoms of the wells were recorded every 15 min. The red curve is the control siRNA transfected cells, the green curve is p97/VCP siRNA transfected cells and the blue curve is SVIP siRNA transfected cells. (b) Out of minimum three total experiments, each had very similar results, demonstrated by the mean values ± standard deviation of quadruplicate wells. *p < 0.05.

The RTCA assay indicated that the invasion and migration capacity of PANC-1 and MIA PaCa-2 cells decreased following transfection with p97/VCP siRNA (Figures 4 and 5). However, there was an increase in migration and invasion with SVIP siRNA (Figures 4 and 5). As a result, the changes in invasions were not significant, but the changes in migrations were statistically significant. In conclusion, the findings suggest that p97/VCP and SVIP have a function in regulating the invasion and migration of PANC-1 and MIA PaCa-2 cells.

## 4. Discussion

The morbidity and mortality rates of pancreatic cancer are high, with early metastasis, local invasion, and chemoresistance being common traits of pancreas cancer. The majority of patients receive their diagnoses at an advanced stage, which results in a low success rate for surgical resection. A better comprehension of the molecular processes that regulate pancreatic cancer metastasis is critically required to increase survival rates [33–36].

Previous studies undertaking microscopic investigations of MIA PaCa-2 and PANC-1 cells indicate that there are two different morphological types in MIA PaCa-2 (small cells and large cells), whereas there are three different morphological types in PANC-1 (small cells, intermediate cells, and large cells) [37]. Additionally, immunohistochemical analysis showed that E-cadherin is expressed in MIA PaCa-2 but not in PANC-1. Because boosted expression of E-cadherin in different tumor types is related to advanced survival [38], it is thought that the attitude of PANC-1 is more aggressive with bigger metastatic capacity.

The ER participates in a variety of cellular functions, including calcium homeostasis, protein folding and transport, and lipid and protein biosynthesis. ER homeostasis is disrupted by nutritional insufficiency, high metabolic demands, oxidative stress, and calcium imbalance in the tumor microenvironment, resulting in ER stress. The role of the multicomponent, complex ERAD system, which includes the proteins gp78, Derlin-1/2, Hrd1, Sel1L, and p97/VCP, is proteasomal degradation, ubiquitination, and translocation of nonnative proteins. Recent research revealed that the ER-associated protein degradation complex has its own UPR-independent capabilities in addition to functioning as a downstream reply of the unfolded protein response, and that there is crosstalk between ERAD and UPR [39].

Previous studies showed an augmented expression of p97/VCP in different types of human cancer cells. Several studies indicated that p97/VCP be targeted in cancer treatments. Suppression of the function of p97/VCP by using siRNA or specific inhibitors causes apoptosis and activation of caspase in human cancer cells [40–43].

An interacting cofactor known as SVIP regulates p97/VCP, which is involved in the ERAD pathway. It also acts as an endogenous inhibitor [26]. In a recent study, a tumor suppressive function of SVIP was identified, and its expression has been related to increased ER stress and suppression of cancer growth [44]. Moreover, SVIP has been defined as an androgen-regulated gene in glioma and prostate cancer [45,46]. In one experimental study, silencing the expression of the ERAD genes SVIP, gp78, and Hrd1 reduced the malignant transformation and migration of LNCaP prostate cancer cells. These results showed that androgens impact the expressions of ERAD components, and promote their proteolytic activity, which is positively related to prostate tumor development [46].

As SVIP was identified to be related to the tumorigenesis of breast, glioma, and prostate cancer, we chose to study SVIP expression with a focus on pancreatic cancer. Previous studies have shown that SVIP is expressed in the adrenal gland at a coherently high level in the course of postnatal development. Therefore, the modulation of SVIP expression is indicated to change not only hormone output and steroidogenic gene expression levels in the cells but also an expression of genes necessary for de novo cholesterol biosynthesis, trafficking, uptake [47].

In this study, SVIP and p97/VCP protein expression have been identified in PANC-1 and MIA PaCa-2 cells by the Western blot technique. Additionally, the cancer cells were transfected with control si, p97/VCPsi, and SVIPsi RNA to detect if the protein expression of p97/VCP and SVIP were impacted in response to this silencing. At 48 h after transfection, the SVIP expression was decreased in the SVIP-depleted cells compared to cells treated with control siRNA, as demonstrated by Western blotting. Furthermore, p97/VCP expression was reduced in the p97/VCP-depleted cells compared to the control siRNA.

Conventional assays such as MTT can be used to monitor the invasion, migration, and viability of cancer cell lines. The use of xCELLigence real-time cell analysis (RTCA) allows the invasive capacity of bladder cancer cells to be accurately assessed and monitored. [48,49]. Because RTCA techniques are more sensitive, they allow better diagnosis of new molecules included in the development of metastatic lesions and the progression of bladder urothelial cancer. [50,51].

In an experimental study, suppression of SVIP increased the proliferation of ZR-75-1 cells and p53 wt MCF-7, but not of SK-BR-3 and p53 mutant T47D cells; it also boosted the migration capacity of both types of cell lines. These findings, together with in silico data analysis, demonstrated the SVIP function and differential expression in breast cancer cells [52].

Another study indicated that the inhibition of VCP suppressed the migration and proliferation of NSCLC cell lines and sped up apoptosis [53]. Similarly, another study evaluated the effects of VCP on invasive and migratory capacity of HCC cell lines. The wound-healing assay showed that an Huh7 cell line transfected with VCP siRNA demonstrated reduced migratory potential compared to cells transfected with nonspecific siRNA. Similar findings were observed in the MHCC-LM3 cell line, where overexpression of VCP accelerated the speed of migration [54].

In a study using p97/VCP siRNAs in human osteosarcoma cell lines, it was determined that migration rate and invaded cell count both decreased in cells where VCP was downregulated, compared with those transfected with Neg-LV after 24 h of transfection [55]. In another study, VCP knockdown was demonstrated to inhibit invasion, chemoresistance, and cell proliferation, and stimulate apoptosis in HCT116 CRC (colorectal cancer) cells [56]. In a study conducted with lymphoma cell lines, after transfection with p97/VCP, siRNA supported the apoptosis of cells. It was also observed that VCP knockdown by siVCP reduced the invasion ability of BCL cells compared with the negative control group [57].

Increased cell migration and invasion are key to the progression of metastatic tumors [58]. This study investigated the suppressive impacts of p97/VCPsi and SVIPsi on cell migration and invasion using RTCA techniques. With regard to p97/VCP, the findings of this investigation are consistent with earlier results determined in various types of human cancers, such as lung cancer, and liver cancer [53,54]. Similarly, with regard to SVIP, the findings of the current study are consistent with earlier results in human breast cancer [52].

This study revealed that SVIP silencing increased the migration and invasion potential of PANC-1 and MIA PaCa-2 cells while p97/VCP silencing decreased the migration and invasion ability of these cells. Furthermore, invasion and migration of both cells were affected by the suppression of p97/VCP and SVIP, indicating that pancreatic cancer cell lines with lower SVIP expression may have higher invasion and migration ability while those with lower p97/VCP expression may have lower invasion and migration ability.

## 5. Conclusion

This work has shown protein expressions and cellular localizations of SVIP and p97/VCP in MIA PaCa-2 and PANC-1 cells. This work also demonstrated, for the first time, SVIP expression in PANC-1 and MIA PaCa-2 cells. When both cell types were transfected with p97/VCPsi and SVIPsi, it impacted the protein expressions as well as the invasion and migration capacity of the cells. Furthermore, as p97/VCP and SVIP are important regulators of proteostasis, their higher expression in pancreatic cancer compared to normal tissues indicates that p97/VCP and SVIP could have a function in pancreatic tumorigenesis.
